# Hyperglycemia Promotes K-Ras-Induced Lung Tumorigenesis through BASCs Amplification

**DOI:** 10.1371/journal.pone.0105550

**Published:** 2014-08-21

**Authors:** Carla Micucci, Silvia Orciari, Alfonso Catalano

**Affiliations:** Department of Clinical and Molecular Sciences, Polytechnic University of Marche, School of Medicine, Ancona, Italy; H. Lee Moffitt Cancer Center & Research Institute, United States of America

## Abstract

Oncogenic K-Ras represents the most common molecular change in human lung adenocarcinomas, the major histologic subtype of non–small cell lung cancer (NSCLC). The presence of K-Ras mutation is associated with a poor prognosis, but no effective treatment strategies are available for K-Ras -mutant NSCLC. Epidemiological studies report higher lung cancer mortality rates in patients with type 2 diabetes. Here, we use a mouse model of K-Ras-mediated lung cancer on a background of chronic hyperglycemia to determine whether elevated circulating glycemic levels could influence oncogenic K-Ras-mediated tumor development. Inducible oncogenic K-Ras mouse model was treated with subtoxic doses of streptozotocin (STZ) to induce chronic hyperglycemia. We observed increased tumor mass and higher grade of malignancy in STZ treated diabetic mice analyzed at 4, 12 and 24 weeks, suggesting that oncogenic K-Ras increased lung tumorigenesis in hyperglycemic condition. This promoting effect is achieved by expansion of tumor-initiating lung bronchio-alveolar stem cells (BASCs) in bronchio-alveolar duct junction, indicating a role of hyperglycemia in the activity of K-Ras-transformed putative lung stem cells. Notably, after oncogene K-Ras activation, BASCs show upregulation of the glucose transporter (Glut1/Slc2a1), considered as an important player of the active control of tumor cell metabolism by oncogenic K-Ras. Our novel findings suggest that anti-hyperglycemic drugs, such as metformin, may act as therapeutic agent to restrict lung neoplasia promotion and progression.

## Introduction

Lung cancer is the leading cause of cancer mortality in the United States and worldwide: the 5-year relative survival rate has not been significantly improved during the last 30 years and remained limited to about 15%. Surgery remains the only curative option for most of the patients with localized disease for whom systemic treatment usually fails to reach a complete and durable response, mostly in the advanced level of this disease [1]. Four histologic types comprise the majority of lung cancers that include Small Cell Lung Cancer (SCLC-20%) and three Non-Small Cell Lung Cancer (NSCLC-80%) types: adenocarcinomas (AC), squamous-cell carcinoma (SCC) and large cell carcinoma. Among those, AC is the most frequent subtype and oncogenic K-Ras is expressed in about 30% of AC [2]. A growing body of evidence indicates that K-Ras mutation is important in the initiation of lung adenocarcinoma development. Indeed, K-Ras mutation has been identified in atypical adenomatous hyperplasia (AAH) lesions, which are thought to precede the development of lung adenocarcinoma [3], [4]. However, beside the genetic factors, other factors are known to influence the high incidence and mortality of lung cancer. Among them, environmental factors, particularly diabetes, may play a role in development and progression of lung cancer. In a recent study, conducted by the independent academic coordinating center of the Emerging Risk Factors Collaboration (ERFC), the hazard ratio for death in lung cancer was found moderately higher among participants with diabetes compared to those without diabetes [5].

Diabetes is a multisystemic disorder. It is unclear whether diabetes has a direct effect due to hyperglycemia that refers to an elevated level of glucose in the bloodstream or an indirect one due to insulin resistance, hyperinsulinemia or other shared risk factors (e.g. obesity) [4], [6]. Some mechanisms have been hypothesized to understand why the impaired fasting glucose concentration is a risk factor for many cancers. An excess level of glucose can lead to accumulation of glycation end-products in the cells that causes the degenerative changes in cell metabolism leading to the carcinogenic mutation [7]–[9]. Moreover high glucose concentration can better sustain the intensive proliferation of typical cancer cells [10], [11].

Candidate lung cancer progenitor cells (bronchio-alveolar stem cells or BASCs) have been identified in murine models of K-Ras induced lung cancer. BASCs are located at the terminal bronchioles, and have self-renew capacity, undergo multi-linage differentiation [12] and, also, have a typical stem cell feature of being double positive for two epithelial markers: Clara cell specific antigen (CC10) and surfactant protein (SP-C) typical of bronchiolar and alveolar cells. After intra-peritoneal injection of 4-hydroxytamoxifen (4-OHT), which induces lung adenocarcinoma due to activation of a conditional oncogenic K-Ras allele [13], BASCs rapidly proliferate prior to the development of histological abnormalities. The BASCs in this mouse model contain the K-Ras^V12^ mutation identical to that found in some human lung tumors [12].

Our study is focused on better understanding the effect of high glucose concentration on lung carcinogenesis. Taking advantage from the K-Ras^V12^ mouse model, we demonstrate that the hyperglycemic status accelerates tumor formation by BASCs amplification.

## Materials and Methods

### Reagents

Antibodies purchased for these studies include anti-Glut2 (NBP2-22218) (Novus Biologicals), anti-insulin (LS-B2526) (LifeSpan BioSciences), anti-Prosurfactant Protein C (AB3786) (Millipore), CC10 (T-18): sc-9772 (Santa Cruz Biotechnology), anti-Glut1 (07-1401) (Millipore). Other purchased reagents include biotinylated anti-rabbit IgG (BA-1000), biotinylated anti-goat IgG (BA-5000) and Vectastain ABC Kit (Vector Laboratories), Texas Red Conjugated goat Anti-Rabbit IgG (31506) (Thermo Scientific).

### Animals

The genetic model used was the K-Ras (+/LSLG12Vgeo); RERTn (ert/ert) mice described by Guerra *et al* [13]. The mouse model was a gift from M. Barbacid, director of the Spanish National Cancer Research Center (CNIO). Mice were maintained on a 12 h light/dark cycle. The studies of transgenic mice were reviewed and approved by the committee “Comitato Etico per la Sperimentazione Animale (C.E.S.A.)” of Polytechnic University of Marche, Ancona, Italy.

### Experimental groups and protocols

For induction of K-Ras^V12^ expression, mice of, at least, 4 weeks of age were injected intraperitoneally with 4-hydroxy-tamoxifen (4-OHT) as previously reported [13]. Mice were divided into four groups with at least three mice per group: wild type (**Untreated-WT**), K-Ras (+/LSLG12Vgeo); RERTn (ert/ert) treated with 4-OHT (**Untreated-K-Ras^V12^**), K-Ras (+/LSLG12Vgeo); RERTn (ert/ert) treated with 4-OHT and with streptozotocin (**STZ-K-Ras^V12^**), and wild type treated with streptozotocin (**STZ-WT**). To activate the K-Ras oncogene in untreated-K-Ras^V12^ group, K-Ras (+/LSLG12Vgeo); RERTn (ert/ert) mice were treated with 4-OHT (0,1 mg 3 times a week for 2 weeks) [13]; to obtain the STZ-K-Ras^V12^ mice, after the 4-OHT treatment, we started with the intraperitoneal administration of 40 mg/kg of streptozotocin dissolved in sodium citrate buffer (pH 4.5) for 5 consecutive days as reported in **Figure S1** [15]. The control groups consist in wild type mice treated with streptozotocin, to create the STZ-WT, and wild type mice without any treatment. Blood glucose was measured before treatment with STZ and then after 4, 8, 12 and 18 weeks in all mice groups, after 3 hours fast (**Figure S1**). Tail-vein glucose was measured between 10 A.M. and 12:00 P.M., and mice were considered diabetic when blood glucose level were >200 mg/dl in two consecutive measurements using the GLUCOCARD G+ meter (A. Menarini Diagnostics s.r.l, Firenze, Italy)

### Tissue collection and immunohistochemical analysis

Mice were sacrificed at different time points. After 4 weeks from streptozotocin treatment (and it means 5 weeks after 4-OHT treatment) also called early time collection, after 12 weeks and after 24 weeks also called late time collection, the mice were euthanized and we collected lung, pancreas, kidney and liver. An half or a part of those organs was used to perform the X-Gal staining [14] and the other part was formalin-fixed and paraffin-embedded. For immunohistochemical staining, 4 µm sections were sequentially incubated in Xylene (5 min twice), 100% ethanol (5 min), 95% ethanol (5 min), 70% ethanol (5 min) and then in water. After that, the sections were antigen-retrieved using citrate buffer (ph 6.0 Dako) in a steamer for 45 min and then cooled to ambient temperature. Sections were then washed with PBS and quenched with 3% hydrogen peroxide in PBS for 10 min, blocked for avidin/biotin activity and incubated with primary antibody as follows: for Glut2, Insulin, SP-C and CC10, sections were incubated at 4°C overnight; for Glut1, sections were incubated with primary antibody for 45 min at room temperature. After primary antibody incubation, sections were washed with PBS and then incubated with biotinylated anti-rabbit or biotinylated anti-goat IgG (1∶200) for 30 min, then washed and incubated with ABC-horseradish peroxidase. Antibody binding was visualized with diaminobenzidine and counterstained with hematoxylin. Finally, sections were dehydrated through graded alcohol, cleared in xylene, and coverslipped.

### β-Gal staining for tissues and cells

Cells and tissues were fixed with Glutaraldehyde solution for 7 minutes and for 60 minutes respectively and washed 3–4 times with PBS for 5 minutes each and stain with Staining solution: Na_2_HPO_4_ 80 mM, NaH_2_PO_4_ 20 mM, MgCl_2_ 1,3 mM, K_3_Fe (CN)_6_ 3 mM, K_6_Fe (CN)_6_ 3 mM, X-Gal 1 mg/ml for 24 hours. Both cells and tissues are then paraffin-embedded.

### BASC cells in high-glucose condition

Cells derived from K-Ras (+/LSLG12Vgeo); RERTn (ert/ert) mice were plated in suspension to select BASC cells. After first generation spheres, cells were dissociated mechanically and enzymatically with trypsin and plated in adherent condition at 12000 cells per well in a 24 multi-well. The medium used was DMEM supplemented with BPE 1%, EGF (20 ng/ml), B27 and FBS 1%. The culture was divided into four conditions, resembling the *in vivo* situations. An half of the cells were cultured in the medium described above and a part was treated with 0,6 µM of 4-OHT; the second half was plated in medium supplemented with 30 mM of glucose (high glucose) and a part was treated with 0,6 µM of 4-OHT as well. The cell count was performed after 5 days of culture, in three wells per condition, using the Scepter Cell Counter (EMD Millipore).

### Statistical analysis

The data were expressed as mean ± SEM (standard error of mean). Statistical differences between groups were analyzed using Student's *t*-test. The difference was considered statistically significant at *p* value <0.05. To obtain comparable results, data of at least three mice from each group were used for statistical analysis.

## Results

### Phenotype of hyperglycemic and euglycemic K-Ras^V12^ mice

**Figure 1:**
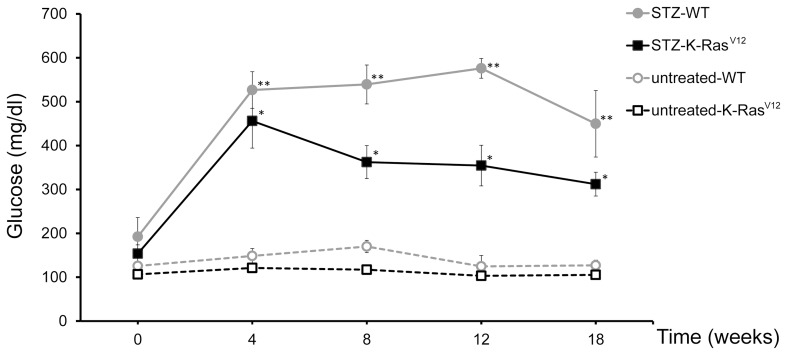
Average blood glucose levels in WT and K-Ras^V12^ mice before and after STZ treatment. Tail-vein glucose was measured after 3 hours of fast. Mean ± standard error of mean; *p<0.05 *versus* untreated-K-Ras^V12^; **p<0.05 *versus* untreated-WT (Student's *t*-test).

**Figure 2:**
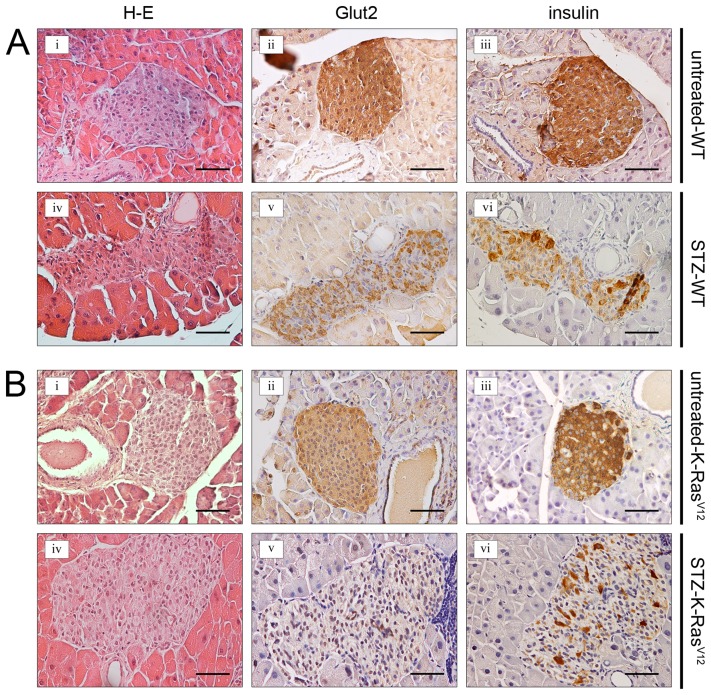
Histology and immunostaining of pancreas in treated and untreated mice with STZ. A. (**i, iv**) H-E of a pancreatic islet in untreated and STZ-WT mice; (**ii, v**) and (**iii,vi**) reduction of Glut2 and insulin expression in STZ-WT mice compared to control mice. **B.** (**i, iv**) H–E of a pancreatic islet in untreated and STZ-K-Ras^V12^ mice; (**ii, v**) and (**iii,vi**) reduction of Glut2 and insulin expression in STZ-K-Ras^V12^ mice compared to untreated K-Ras^V12^ control mice. Magnification 200×. Calibration bar: 50 µm.

STZ induced diabetes is a well-documented model of experimental diabetes [15], [16]. Administration of multiple low doses of STZ (40 mg/kg×5 day, i.p.) produces significant hyperglycemia from 4 weeks in both WT mice and K-Ras^V12^ mice, compared with control groups untreated-WT and untreated-K-Ras^V12^, respectively (**Figure 1**). The animals maintained high glucose level up to 18 weeks. No statistical differences in the diabetic state were observed between untreated-WT and untreated-K-Ras^V12^ mice (**Figure 1**). In a separate experiment, STZ treated and untreated mice were killed at 12 weeks for histological and immunohistochemical analysis of pancreas. For WT and K-Ras^V12^ mice, the architecture of islets after STZ treatment was largely disrupted (**Figure 2A** **-iv, 2B-iv**), weaker Glut2 positive-cells were observed (**Figure 2A** **-v, 2B-v**) and the intensity of insulin staining was reduced (**Figure 2A** **-vi, 2B-vi**). On the other hand, a normal shape of pancreatic islet and a normal expression of both Glut2 and insulin were found in untreated control WT and K-Ras^V12^ mice, represented in **Figure 2A** **-i, ii, iii** and **Figure 2B** **-i, ii, iii**, respectively [17].

### Chronic hyperglycemia increases lung tumorigenesis in K-RasV12 mice

**Figure 3:**
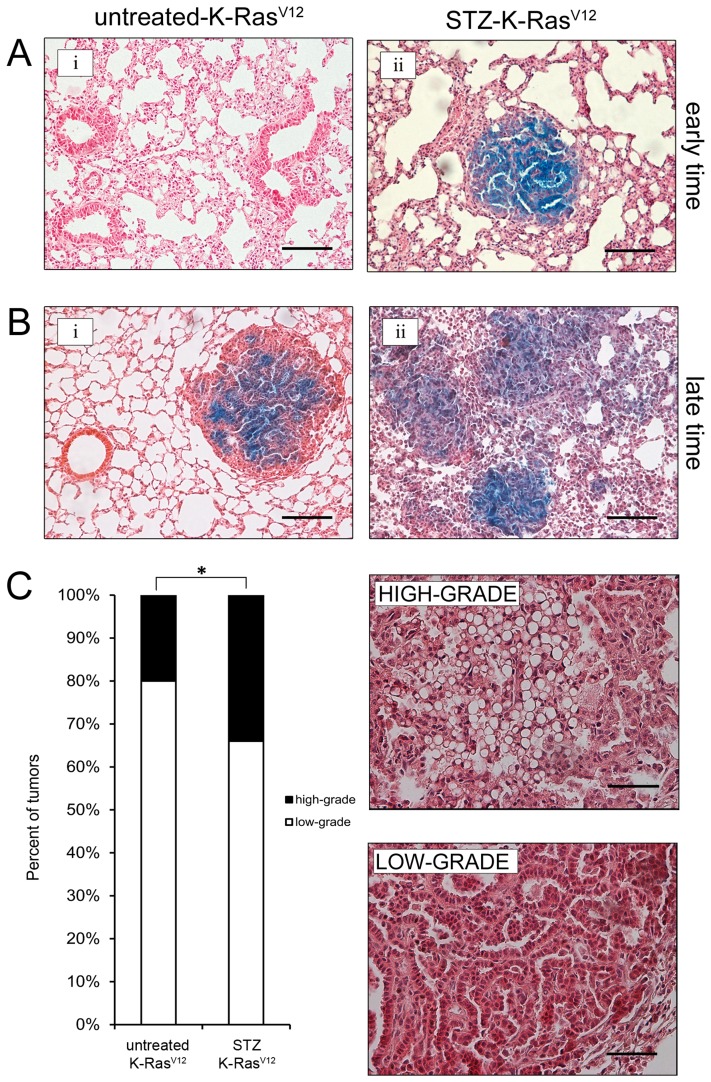
Early and late time representation of tumor formation and progression, respectively. **A**. Early time, after 4 weeks from STZ treatment, no tumor formation in untreated-K-Ras^V12^ mice (**i**) and some tumors in K-Ras^V12^ mice treated with STZ (**ii**). **B**. Late time, after 24 weeks from STZ treatment, well defined masses in untreated-K-Ras^V12^ mice (**i**) and several multifocal tumor distribution in K-Ras^V12^ treated with STZ (**ii**). **C**. Assessment of tumors at low and high grade in untreated-K-Ras^V12^ and STZ- K-Ras^V12^. **Left**: percent of low (white bar) and high (black bar) tumors in STZ- K-Ras^V12^ and untreated-K-Ras^V12^ mice (mean ± SEM; *, *p*<0.01 by X^2^ test). **Right**: representative H-E of low and high lung tumor grade. H-E, magnification 100×. Calibration bar: 100 µm.

**Figure 4:**
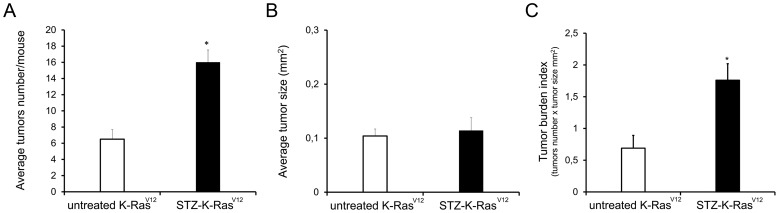
Assessment of tumor development and progression of lung tumors in STZ-K-Ras^V12^ mice compared to untreated-K-Ras^V12^ ones. **A.** Average number of tumors that developed per mouse in STZ-K-Ras^V12^ (black bar) compared to untreated-K-Ras^V12^ (white bar) mice. **B.** Average tumor size (mm^2^) in STZ-K-Ras^V12^ (black bar) and in untreated-K-Ras^V12^ mice (white bar). **C.** Expression of neoplastic tissue with the tumor burden index which considers average number of tumors per mice and average size. Data are presented as mean ± SEM, *p<0.05 (Student's *t*-test).

To assess the role of hyperglycemia in oncogenic K-Ras-initiated lung tumorigenesis, mice were killed and examined for lung tumors. No visible lesions were found during the period of observation in untreated-WT and STZ-WT mice (data not shown). Immunohistochemical examination for the earliest detectable lesions (after 4 weeks from STZ treatment) revealed the presence of few small lesions in the STZ-K-Ras^V12^ mice, while no adenoma were observed in untreated-K-Ras^V12^ mice (**Figure 3A** **i-ii**). At 24 weeks (late time), we noticed some masses in untreated-K-Ras^V12^ mice, with a well-defined distribution, in accordance with the mouse model [13], [18] (**Figure 3B** **-i**). In contrast, several masses with multifocal distribution were present in K-Ras^V12^ mice treated with STZ (**Figure 3B** **-ii**). Furthermore, we observed frequently higher grade adenocarcinomas in STZ-K-Ras^V12^ mice than untreated-K-Ras^V12^ group (34±0.3% vs 20±1.2% respectively; mean ± SEM; *p*<0.01, **Figure 3C**). Consistently, quantitative analysis of visible tumors revealed, at late time, a significant increase of lung lesions in STZ-K-Ras^V12^ mice compared to untreated- K-Ras^V12^ ones (16±1.5 vs 6.5±1.2; *p*<0.05; **Figure 4A**). The tumor size of STZ-K-Ras^V12^ mice was similar to the untreated K-Ras^V12^ (**Figure 4B**). The total amount of cancer cells spread, all over the lung, indicated as tumor burden index, is two fold higher in STZ-K-Ras^V12^ mice than untreated K-Ras^V12^ ones (**Figure 4C**). All together, these data indicate that chronic hyperglycemia promotes tumor initiation and progression during K-Ras^V12^-driven tumorigenesis.

### Hyperglycemia promotes K-Ras-mediated bronchio-alveolar stem cell expansion

**Figure 5:**
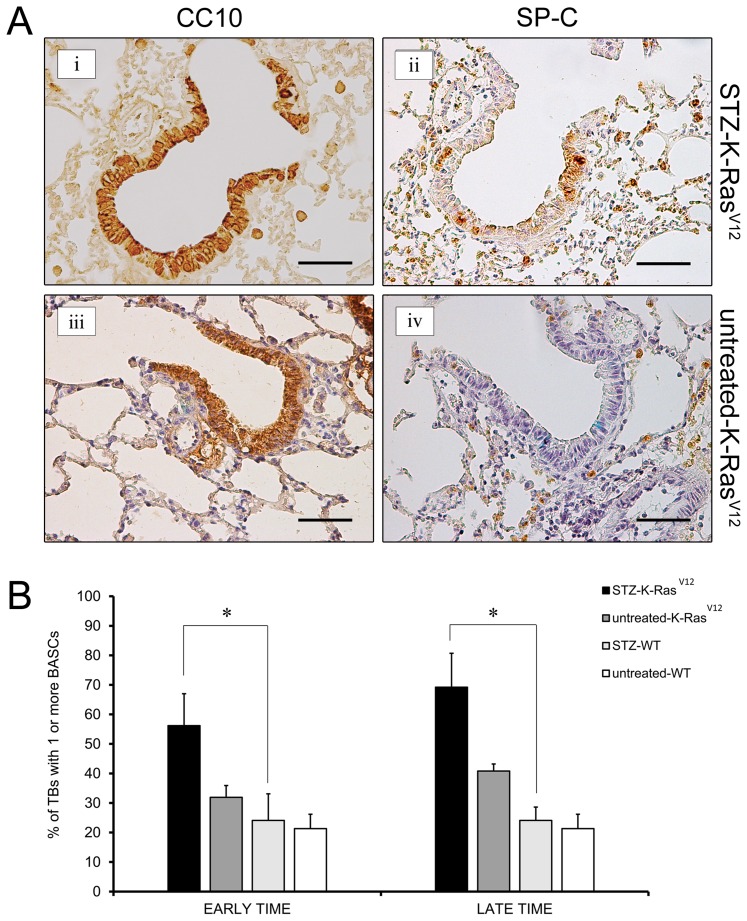
Analysis of BASCs expansion. **A.** Immunohistochemical analysis of CC10 and SP-C in terminal bronchioles of STZ-K-Ras^V12^ (**i, ii**) and untreated-K-Ras^V12^ (**iii, iv**) mice. Magnification 200×. Calibration bar: 50 µm. **B.** Bar graph indicates percentage of terminal bronchioles with 1 or more BASCs at early and late time (STZ-K-Ras^V12^ *vs* STZ-WT: *p<0,01 by X^2^ test).

The fact that the STZ- K-Ras^V12^ mice develop more lung tumors, yet at early time of our observation, suggests that hyperglycemia could influence the initial step of K-Ras^V12^-induced lung tumorigenesis. The clonal expansion of bronchio-alveolar stem cells (BASCs), putative regional stem cells that reside at the terminal bronchioles is thought to be a key event in early step of lung tumor development, at least in this mouse model [13]. Therefore, we assessed whether the presence of high glucose affects the expansion of BASCs expressing oncogenic K-Ras, after 4-OHT injection. To this end, we identified and quantified BASCs in paraffin sections of mouse lungs, by immunohistochemical staining in sequential sections for surfactant protein c (SP-C) and the Clara cell-specific protein (CC10). Within the terminal bronchioles, in STZ-K-Ras^V12^ and untreated-K-Ras^V12^ mice, columnar epithelial cells showed a typical CC10 staining (**Figure 5A** **-i, 5A-iii**). At the bronchio-alveolar duct junction, adjacent the alveolar space, occasional epithelial cells were, also, positive for SP-C staining (**Figure 5A** **-ii, 5A-iv**). SP-C positive cells appeared to be strongly increased in hyperglycemic respect to euglycemic K-Ras^V12^ mice. CC10 and SP-C immunostainings were, also, carried out on lung samples from STZ-WT and untreated-WT mice, showing similar CC10 positive cell distribution, but few sporadic or no cells positive for SP-C (data not shown). Quantitive analysis of lung tissue sections, at early and late time, revealed an increase number of BASCs in STZ-K-Ras^V12^ mice compared to other groups (**Figure 5B**). Thus the hyperglycemic status enhances BASCs amplification particularly when K-Ras^V12^ is activated. Consistently with BASCs expansion as early event in lung tumorigenesis *in vivo*, we observed a positive effect of hyperglycemia on oncogenic K-Ras induced proliferation of BASC cultures. Indeed, BASC-like cells derived from K-Ras (+/LSLG12Vgeo); RERTn (ert/ert) mice, exposed to high glucose media and treated with 4-OHT, underwent a low, but reproducible, increase of about 20% in cell number, compared to control cultures (**Figure S2**).

### K-Ras-mutated BASCs are sensitive to hyperglycemia due to Glut1 expression

**Figure 6:**
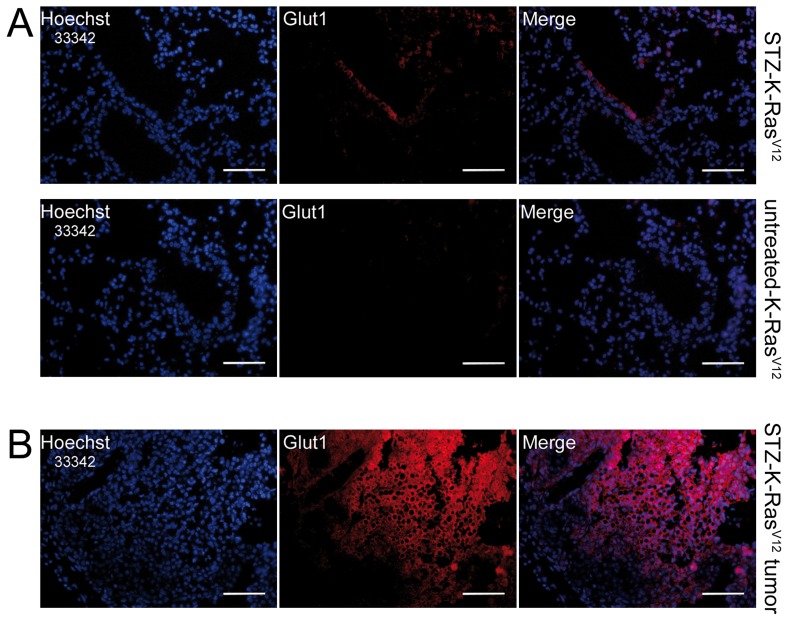
Immunofluorescent staining of tissue sections to detect the Glut1 expression (red). **A.** Terminal bronchioles of both STZ-K-Ras^V12^ and untreated-K-Ras^V12^ mice; **B.** Tumoral masses of STZ-K-Ras^V12^ mice. Magnification 100×. Calibration bar: 100 µm.

**Figure 7:**
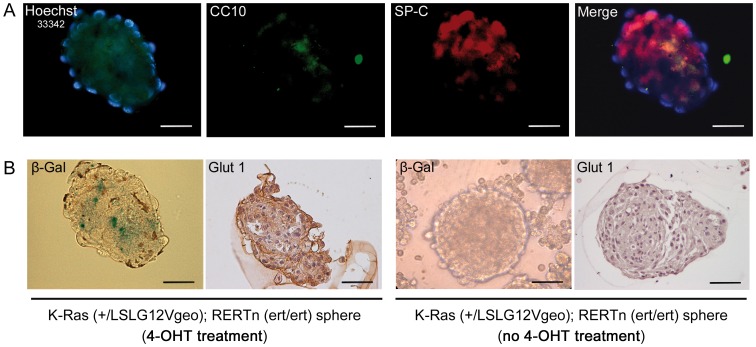
Oncogenic K-Ras induces Glut1 expression in BASCs. **A.** Immunofluorescent analysis of CC10 (green) and SP-C (red) dual positive BASCs of K-Ras (+/LSLG12Vgeo); RERTn (ert/ert) mice after 4-OHT treatment *in vitro*. **B.** Spheres from K-Ras (+/LSLG12Vgeo); RERTn (ert/ert) mice treated with 4-OHT *in vitro* show β-Gal staining and Glut1 expression compared with no Glut1 staining in control spheres from K-Ras (+/LSLG12Vgeo); RERTn (ert/ert) mice not treated with 4-OHT. Magnification 200×. Calibration bar: 50 µm.

Glucose is the major source of energy for cells and glucose transport 1 (Glut1) is the most common glucose transporter. Glut1 has been found to be abberrantly expressed in K-Ras expressing cells [19]–[22], suggesting that mutated BASCs exhibit enhanced expansion and promote tumor formation in high glucose conditions owing to enhanced glucose uptake and glycolysis. Accordingly, we found that expression of Glut1 protein was markedly higher in terminal buds of STZ-K-Ras^V12^ compared to untreated-K-Ras^V12^ mice (**Figure 6A**) or STZ-WT (data not shown). As expected the Glut1 protein was found in tumor masses from STZ-K-Ras^V12^ (**Figure 6B**). To further test the specificity of the up-regulation of Glut1, BASC-like cells were isolated using the colony formation assay from K-Ras (+/LSLG12Vgeo); RERTn (ert/ert) mice. After 4-OHT treatment *in vitro*, we showed BASC-like cells as double-positive sub-population within spheres, using immunofluorescence labeling with CC10 and SP-C antibodies (**Figure 7A**). In those spheres we verified the K-Ras oncogene activation by β-Gal staining, taking advantage of the reporter gene in the mouse model (**Figure 7B**) and we noticed a Glut1 up regulation if compared to the control spheres which were not treated with 4-OHT and not positive for β-Gal staining (**Figure 7B**). Therefore, oncogene K-Ras activation induces Glut1 expression in BASCs cells, promoting glucose affinity, and hyperglycemia increases BASCs cell autonomous growth in Glut1 positive BASCs.

## Discussion

Numerous epidemiologic studies suggest that diabetes may significantly increase mortality in patients with cancers, including lung cancer [5]. However, metformin, the most-widely used drug for type-2 diabetes, has been shown to have anti-neoplastic activity both *in vitro* and *in vivo* in different studies [23]. Recent animal studies show that treating aggressive lung cancer with metformin along with radiation and chemotherapy may slow down tumor growth and recurrence [24]. Although these data clearly suggest a direct role of the diabetic *milieu* in tumor development, these studies do not dissect a pathophysiologic mechanism underlying this phenomenon. A critical question is whether the impact of diabetes on cancer development is due to shared risk factors (obesity, poor diet, physical inactivity and aging) or whether diabetes itself and the specific metabolic derangements, typical of diabetes (e.g., hyperglycemia, insulin resistance, hyperinsulinemia), have a tumor-promoting activity for some types of cancer. Here, we investigated in a mouse model whether hyperglycemia, specifically, affects the growth and progression of lung cancer.

To this end, we used STZ to induce hyperglycemia in a mouse model of K-Ras-driven lung adenocarcinoma. As previously reported, STZ treatment resulted in apoptosis of pancreatic β-cells and consequent inflamatory response contributed and susteined injury [25]. The resulting reduction of insulin levels (**Figure 2**) triggered hyperglycemia, effectively mimicking type 1 or late stages of type 2 diabetes. We showed that in K-Ras mice the hyperglycemic state is sufficient to accelerat lung cancer development. Carcinogenesis is a complex process. Normal cells must undergo multiple genetic “hits” before the full neoplastic phenotype of growth, invasion, and metastasis occurs. This process of malignant transformation can be divided into multiple steps including: initiation (irreversible first step toward cancer), promotion (stimulation of the growth of initiated cells), and progression (development of a more aggressive phenotype of promoted cells). Factors that affect one or more steps of this pathway could be associated with cancer incidence or mortality. Our results indicate that elevated circulating glucose levels in K-Ras-driven lung tumorigenesis play a role as tumor promoter and progressor. Indeed, K-Ras-driven tumors exposed to hyperglycemia *in vivo* grew faster than euglycemic hosts (at early time we, yet, observed tumor mass in STZ-treated mice, **Figure 3A–B**) and showed a more malignant growth behavior (**Figure 3C**). Moreover, our current study provides compelling evidence that hyperglycemia, after activation of oncogenic K-Ras, exerts its pro-tumorigenic effects, at least in part, by maintaining a sub-population of cancer cells, namely tumor-initiating lung bronchio-alveolar stem cells (BASCs). Though it is still unclear whether BASCs represent regional lung stem cells, strong circumstantial evidence indicates that they are involved in tumor initiation in the mouse lung. BASCs undergo expansion and transformation in response to K-Ras activation [12]. Genetic and/or pharmacological disruption of multiple key oncogenic pathway genes involved in K-Ras-mediated tumorigenesis, lead to inhibition of BASC expansion and K-Ras-mediated tumor formation *in vivo* [26]. Human lung adenocarcinomas, which frequently harbor K-Ras mutation, often develop at the bronchio-alveolar duct junction and display either bronchial or alveolar airway differentiation, or both [27], [28], suggesting that some of these tumors may have originated from BASC-like cells.

On the other hand, K-Ras oncogene is known to induce aerobic glycolysis [29]. Recently, it has been shown that K-Ras-mutated cells show rapid upregulation of specific glycolytic metabolic enzymes (e.g. the glucose transporter Glut1/Slc2a1) and their pathways prior to any discernible biological impact (e.g., morphological or proliferative changes), a finding consistent with the active control of tumor cell metabolism by oncogenic K-Ras [30]. Our data provide the evidence that the role of hyperglycemia in lung mutated BASCs is cell autonomous. The K-Ras-derived lung tumors show Glut1 staining in tumor cells, with little to no staining in tumor associated-stroma or morphological normal lung epithelium. More importantly, at the bronchio-alveolar duct junction we observed a strong expansion of oncogenic BASCs expressing the glucose transporter Glut1. Thus, while many metabolic changes produced by the tumor microenvironment play prominent roles in the invasive and metastatic properties of lung tumor cells, our data demonstrate that oncogenic K-Ras specifically enhances glycolytic flux in BASCs to support the autonomous growth of these tumor-initiating cells. Our data do not exclude a contributory role of other factors that influence diverse aspects of diabetes (e.g., hyperinsulinemia or chronic inflammation). However, our studies provide new insight into a largely unappreciated role for hyperglycemia in the regulation of tumor-initiating BASCs behavior. In this regard, it is interesting to note that Glut1 expression has been observed to be elevated in oncogenic K-Ras-positive human lung adenocarcinomas [31], suggesting that our results may also be translated in human malignancy.The tumor-promoting activity of hyperglycemia on tumor-initiating BASCs can be mediated by direct and indirect mechanisms. Hyperglycemia induces oxidative stress and ROS (reactive oxygen species) production [29]. It decreases mitochondrial function and suppresses oxidative phosphorylation pushing the cell energy metabolism towards glycolysis (Crabtree effect), and increases mutagenesis [32]. Moreover, hyperglycemia stabilized hypoxia-inducible factor-1 alpha (HIF-1alpha), crucial for the expression of enzymes from glycolytic pathway [33]. Therefore, it is plausible that elevated circulating glucose levels may also result in HIF-1alpha activation *in vivo*. Even hyperglycemia causes persistent epigenetic changes and altered gene expression [34]. The best understood types of epigenetic regulation are DNA methylation and histone posttranslational modification [35]. By epigenetic mechanism, high glucose condition significantly induces histone acetylation, NF-kB activation, and proinflammatory cytokine (e.g., IL-6 and TNF-alpha) release [36]. Although future studies are needed to uncover the specific chromatin events and molecular mechanisms induced by hyperglycemia that influences lung cancer progression, these data further support our hypothesis that the tumor-promoting activity of hyperglycemia can be associated with several aspects of oncogenesis.

Taken together, our data provide compelling evidence that diabetes accelerates lung cancer progression. We hypothesize that due to oncogenic K-Ras activation and increased Glut1 expression, the BASC-like cells are more susceptible to mitogenic and survival signals induced by hyperglicemic *milieu*. Lung carcinogenesis in diabetic patients represents a two-hit phenomenon, with an oncogene (e.g., K-Ras or others) acting as a tumor initiator and hyperglycemia, at least in part, acting as a tumor promoter. The present study has an important clinical relevance and significant public health implications. Early detection and therapeutic correction of hyperglycemia may help to reduce lung cancer morbidity and mortality, particularly in those expressing oncogenic K-Ras. Furthermore, our data provide the rationale for some diabetes medications as chemopreventive agents in those patients with a risk to develop lung cancer (e.g. smokers, etc.).

## Supporting Information

Figure S1 **Schematic representation of experimental protocol: 4-OHT treatment, STZ treatment, glucose measurements and sacrifices at different time points are shown.**
(TIF)

Figure S2 **Effect of high glucose on BASCs proliferation derived from K-Ras (+/LSLG12Vgeo); RERTn (ert/ert) mice.** Cell number of BASCs after five days of culture in basal (no Glucose) or in high glucose medium, in the presence or absence of 4-OHT (mean ± SEM, n = 3). Cell number was determined by Scepter Cell Counter.
(TIF)
