# Supplementary figures and images for: Hyperglycemia Promotes K-Ras-Induced Lung Tumorigenesis through BASCs Amplification
Source: PLoS One. 2014 Aug 21;9(8):e105550. doi: 10.1371/journal.pone.0105550 (PMC4140809; doi:10.1371/journal.pone.0105550)

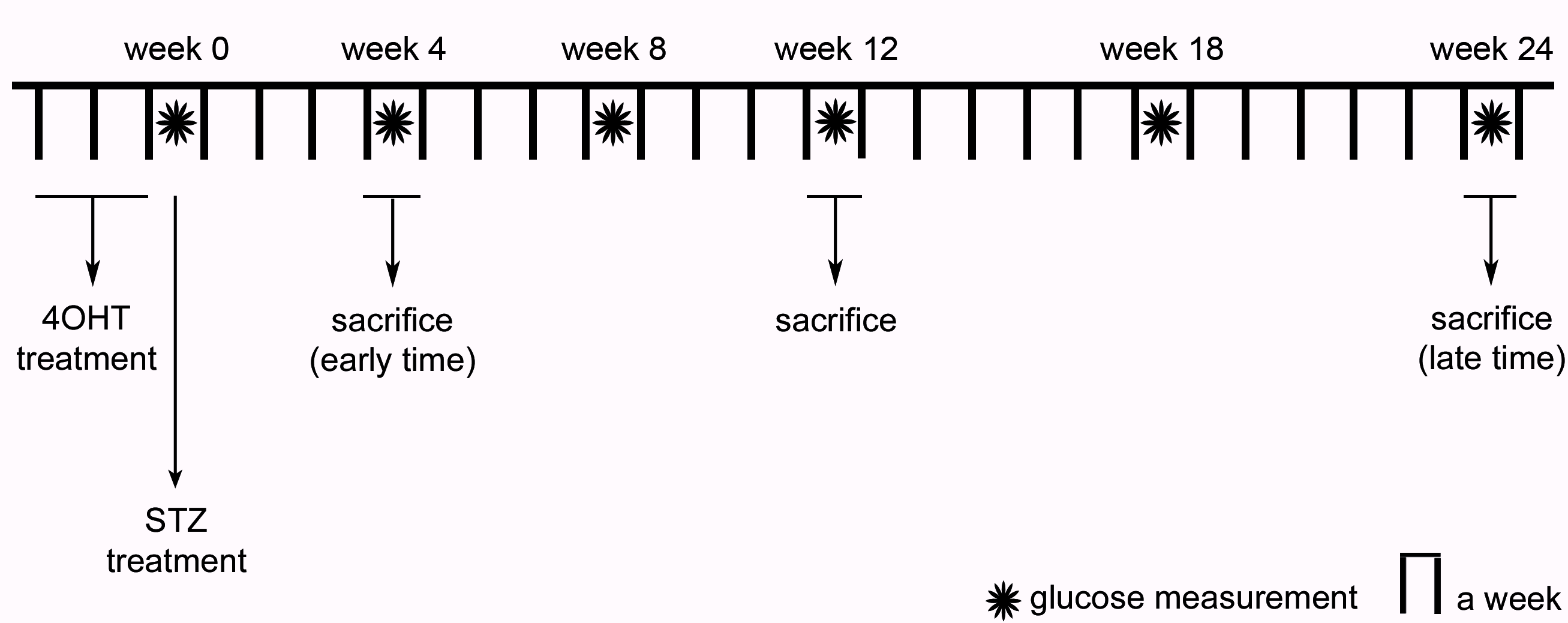

Supplement: Figure S1 — Schematic representation of experimental protocol: 4-OHT treatment, STZ treatment, glucose measurements and sacrifices at different time points are shown. (TIF) [file pone.0105550.s001.tif]

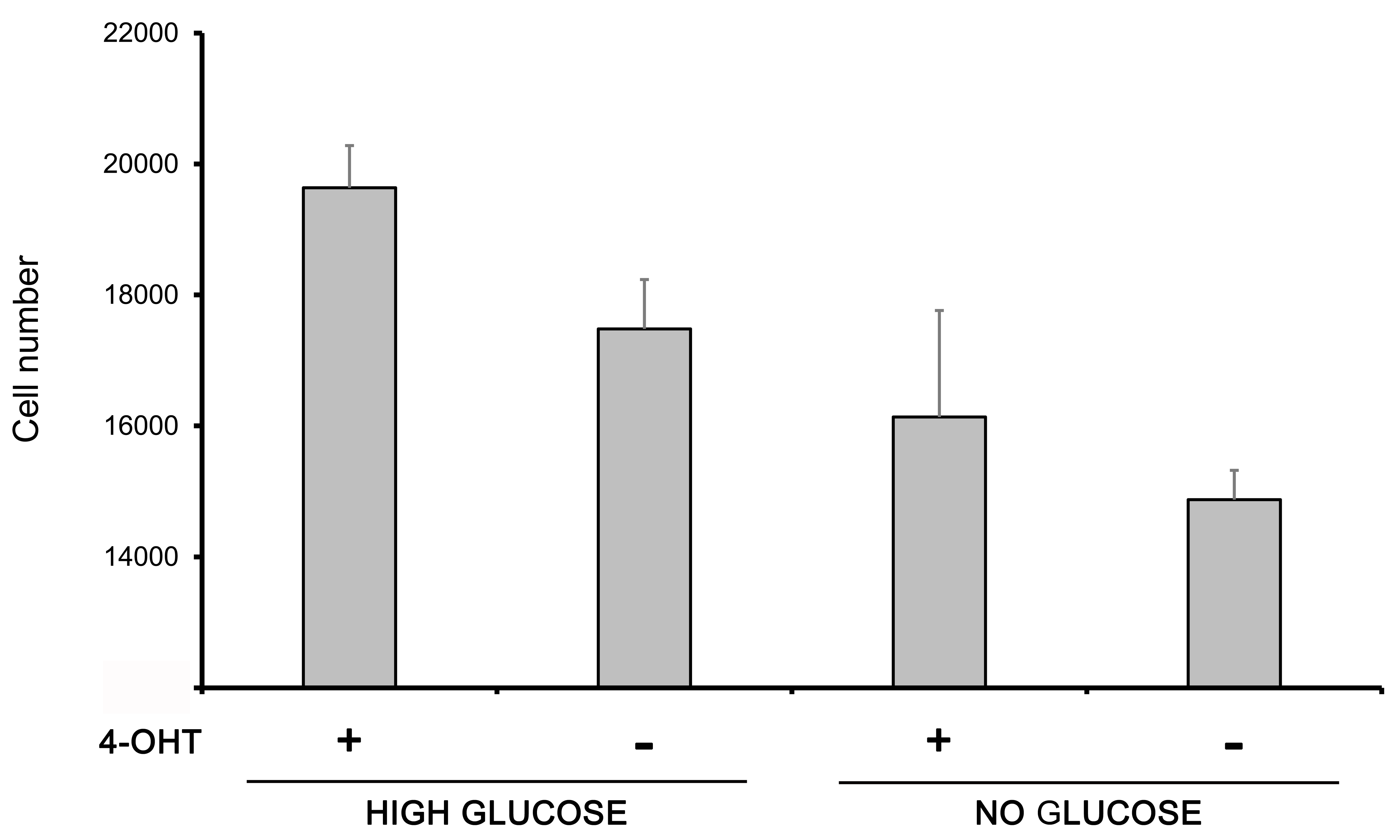

Supplement: Figure S2 — Effect of high glucose on BASCs proliferation derived from K-Ras (+/LSLG12Vgeo); RERTn (ert/ert) mice. Cell number of BASCs after five days of culture in basal (no Glucose) or in high glucose medium, in the presence or absence of 4-OHT (mean ± SEM, n = 3). Cell number was determined by Scepter Cell Counter. (TIF) [file pone.0105550.s002.tif]
